# VIM-Based Dynamic Sparse Grid Approach to Partial Differential Equations

**DOI:** 10.1155/2014/390148

**Published:** 2014-02-27

**Authors:** Shu-Li Mei

**Affiliations:** College of Information and Electrical Engineering, China Agricultural University, P.O. Box 53, East Campus, 17 Qinghua Donglu Road, Haidian District, Beijing 100083, China

## Abstract

Combining the variational iteration method (VIM) with the sparse grid theory, a dynamic sparse grid approach for nonlinear PDEs is proposed in this paper. In this method, a multilevel interpolation operator is constructed based on the sparse grids theory firstly. The operator is based on the linear combination of the basic functions and independent of them. Second, by means of the precise integration method (PIM), the VIM is developed to solve the nonlinear system of ODEs which is obtained from the discretization of the PDEs. In addition, a dynamic choice scheme on both of the inner and external grid points is proposed. It is different from the traditional interval wavelet collocation method in which the choice of both of the inner and external grid points is dynamic. The numerical experiments show that our method is better than the traditional wavelet collocation method, especially in solving the PDEs with the Nuemann boundary conditions.

## 1. Introduction

The sparse representation of functions via a linear combination of a small number of basic functions has recently received a lot of attention in several mathematical fields such as approximation theory as well as signal and image processing [1]. The advantage of the sparse grid approach is that it can be extended to nonsmooth solutions by adaptive refinement methods; that is, it can capture the steep waves appearing in the solution of the PDEs. In fact, the boundary conditions can also be taken as the nonsmooth parts appearing in the solution, especially to the Neumann boundary. Furthermore, it can be generalized from piecewise linear to high-order polynomials. Also, more sophisticated basis functions like interpolets, prewavelets, or wavelets can be used in a straightforward way [2]. In practice, the standard piecewise linear multiscale basis in one dimension, that is, the Faber-Schauder, can be viewed as a scaling function in the wavelet analysis. As an interpolation operator, the basis functions act as the Dirac delta function when operating on itself and its derivatives [3]. So, the interpolation wavelet such as the Shannon wavelet, Shannon-Gabor wavelet, Harr wavelet, and the autocorrelation function of the Daubechies scaling function can be taken as the basis function to construct the sparse grid approach directly.

Faber-Schauder and Haar scaling function do not have the smoothness property, so the function and its derivative to be approximated cannot be represented exactly by them. The autocorrelation function of Daubechies scaling function has been widely used in various numerical methods for PDEs such as the wavelet collocation method and the sparse grids method. The Daubechies scaling functions possess almost all the excellent numerical properties, such as orthogonality, smoothness, and compact support, which are helpful in improving numerical accuracy and efficiency. However, the autocorrelation function of the Daubechies scaling function loses the orthogonality. In addition, Daubechies scaling function has no exact analytical expression. This will bring error to the approximation solution from the Daubechies wavelet numerical method.

Cattani studied the properties of the Shannon wavelet function [4], which possesses many advantages such as orthogonality and is continuous and differentiable. It also has the advantage over the Hermite DAF in that it is an interpolating function, producing matrix equations that have the potential to be relatively sparse. In addition, the second-order approximation of a C^2^-function, based on Shannon wavelet functions, is given [5]. The approximation is compared with the wavelet reconstruction formula and the error of approximation is explicitly computed [6]. The advantages of the Shannon wavelet have been illustrated in solving PDEs in engineering [7], which can avoid the shortcomings of Daubechies wavelet such as the interpolation property. Furthermore, Cattani studied the fractional calculus problems with the Shannon firstly. A perceived disadvantage of the Shannon scaling function is that it tends towards zero quite slowly. The direct consequence of this is that a large number of the nodal values will contribute significantly when calculating the derivatives of the function to be approximated. It is for that reason that Hoffman et al. constructed the Shannon-Gabor wavelet [8] using the Gaussian window function. In some ways it improves the approximation to a Dirac delta function compared with Shannon wavelet. However, the presence of the Gaussian window destroys the orthogonal properties possessed by the Shannon wavelet, effectively worsening the approximation to a Dirac delta function. In order to test the multilevel interpolation operator constructed in this paper, the Faber-Schauder, Shannon scaling functions and the autocorrelation function of the Daubechies scaling function are taken as the basis employed in the multilevel interpolator to discretize the PDEs in the experiments, respectively.

There are many ways to solve the system of nonlinear ODEs, which are obtained from the discretization of the nonlinear PDEs using the multilevel interpolator. Compared with the finite difference method, the retained grid points are sparse and the dimensionality of the system of ODEs is smaller. This is helpful to improve the efficiency, but the small change of the condition number and the smoothness of the function to be approximated can destroy the exactness of the numerical solution obtained by the traditional difference method. The variational iteration method proposed by Inokuti et al. in 1978 [9], which has been developed by He [10, 11] and widely used in various fields [12–14], is able to give the solution of the nonlinear problems in an infinite series usually converging to an accurate solution rapidly. By means of the precise integration method (PIM), VIM has been generated to solve the system of nonlinear ODEs by Mei and Zhang [15]. In fact, both of the PIM and VIM are the analytical method; so, the impact of the system of ODEs on the choice of the sparse grid points can be neglected.

The dynamic choice of the inner grid points relates with the smoothness and the gradient at each point of the solution to be approximated, and the external grid points relates with the boundary conditions. A better choice of the external grid points can restrict the boundary effect effectively. Most of the schemes are based on the extension of the solution function, such as the interval wavelet in the wavelet collocation method [16] and Lagrange multiplier in the sparse grid approaches [17]. In most cases, the smoothness and the gradient around the boundary are variational dynamically such as the Nuemann boundary conditions. The extension method by means of the Lagrange multiplier is not suited to change the external grid points dynamically.

In our approach we want to achieve several goals so that the solution should be sparse and should be a good approximation. First of all is to construct a multilevel interpolation operator with which the adaptive sparse grid approach can be simplified to a linear combination of the interpolation operators. The operator should be independent with the basic functions. So, we can take different basis functions in the interpolation operator to solve different problems. Second goal is to construct an adaptive sparse grid approach by combining the multilevel interpolation operator and the VIM. The last one is to construct a dynamic choice scheme on the external grid points, so that both of the inner and external grid points are dynamic with the development of the solution, especially to PDEs with the Neumann boundary conditions.

## 2. Multilevel Interpolator on Sparse Grids

### 2.1. Interpolating Multiresolution Analysis

Let us start with the interpolating multiresolution analysis [18] that is necessary for a detailed discussion of sparse grids for purposes of interpolation or approximation, respectively. Let *ϕ*(*x*) be any of the interpolating basis function such as the Shannon, Faber-Schauder, scaling functions or the autocorrelation function of the Daubechies scaling function. This mother of all basis functions can be used to generate an arbitrary *ϕ*_*k*_^*j*^(*x*) by dilation and translation; that is

(1) $$\begin{array}{c} \phi_{k}^{j}\left(x\right)=\phi \left(2^{j}x-k\right),\;k=0,1,2,\ldots ,2^{j}. \end{array}$$

It is easy to check that introducing the spaces

(2) $$\begin{array}{c} V^{j}≔\mathrm{span}\langle \phi_{k}^{j},k=0,1,2,\ldots ,2^{j}\rangle \subset L^{2}\left(\mathbb{R}\right). \end{array}$$

For convenience of notation we use the superscript to denote the level of resolution and the subscript to denote the location in physical space. The sequence {*V* ^*j*^} is a multiresolution analysis, which is an increasing sequence of closed subspaces of *L* ^2^(ℝ). We call such a structure an interpolating multiresolution analysis due to the fact that the function *ϕ* verifies what we call interpolation property, that is *ϕ*_*k*_^*j*^(*n*2^−*j*^) = *δ*_*n*,*k*_. We may then define an interpolation operator *I* ^*j*^ : *C* ^0^(0,1) → *V* ^*j*^

(3) $$\begin{array}{c} I^{j}f=\sum_{k=0}^{2^{j}}f\left(x_{k}^{j}\right)\phi_{k}^{j},\;x_{k}^{j}=k2^{-j}. \end{array}$$

It is obvious that *ϕ*_*k*_^*j*^ is just the nodal point basis of the finite-dimensional space *V* ^*j*^. Additionally, we introduce the hierarchical increments *W* ^*j*^ ⊂ *V* ^*j*+1^

(4) $$\begin{array}{c} W^{j}=\mathrm{span}\langle \psi_{k}^{j},k=0,1,2,\ldots ,2^{j}-1\rangle , \end{array}$$

where *ψ*_*k*_^*j*^ = *ϕ*_2*k*+1_^*j*+1^.

Let *y*_*k*_^*j*^ = *x*_2*k*+1_^*j*+1^, we may remark that the function *ψ*_*k*_^*j*^ verifies

(5) $$\begin{array}{c} \psi_{k}^{j}\left(y_{n}^{j}\right)=\delta_{kn},\;\psi_{k}^{j}\left(y_{n}^{j\prime }\right)=0,\;\forall j^{\prime }<j. \end{array}$$

It is obvious that

(6) $$\begin{array}{c} V^{j+1}=V^{j}⨁W^{j}. \end{array}$$

Such multiresolution analysis has been extensively investigated in [9]. According to this theory, any function *f* ∈ *C* ^0^(0,1) can be represented approximately as:

(7) $$\begin{array}{c} f\approx f^{j}=\sum_{k=0}^{2^{j_{0}}}\beta_{k}^{j_{0}}\phi_{k}^{j_{0}}+\sum_{j\geq j_{0}\;}\sum_{k=0}^{2^{j}}\alpha_{k}^{j}\psi_{k}^{j}. \end{array}$$

The coefficients *β*_*k*_^*j*_0_^ and *α*_*k*_^*j*^ are defined as:

(8) $$\begin{array}{c} \beta_{k}^{j_{0}}=f\left(x_{k}^{j_{0}}\right),\;\;\alpha_{k}^{j}=f\left(y_{k}^{j}\right)-I^{j}f\left(y_{k}^{j}\right), \end{array}$$

respectively. This shows that the coefficient *α*_*k*_^*j*^ measures the lack of approximation of *f* by *I* ^*j*^ *f* [19].

### 2.2. Multilevel Interpolation Operator on Sparse Grids

Equation (7) is the approximate representation of function *f*, which is not unique since the set of functions is not linearly independent. In this section, we will try to determine the sparsest representation, that is, a representation with a maximal number of vanishing coefficients among the coefficients {*α*_*k*_^*j*^, *k* = 0,1,…, 2^*j*^,  *j* ∈ *Z*}. The conventional scheme in signal processing, acquiring the entire signal and then compressing it, was questioned by Donoho and Elad [20]. Indeed, this technique uses tremendous resources to acquire often very large signals, just to throw away information during compression. The popular solving scheme is the compressed sensing technique proposed by Donoho [21]. In contrast to it, we try to achieve the same goals by constructing a multilevel interpolation operator via combining the interpolating multiresolution analysis described above and the wavelet transform theory [22].

Let us start with the definition of the interpolation operator

(9) $$\begin{array}{c} u^{J}\left(x\right)=\underset{i\in Z_{\Omega }^{J}}{\sum }I_{i}\left(x\right)u_{i}^{J},\;Z_{\Omega }^{J}:=0,1,2,\ldots ,2^{J}, \end{array}$$

*I*_*i*_(*x*) is the interpolation function. According to the wavelet transform theory, function *u*(*x*) can be expressed approximately as:

(10) $$\begin{array}{c} u^{J}\left(x\right)=\overset{2^{j_{0}}}{\underset{k_{0}=0}{\sum }}u\left(x_{k_{0}}^{j_{0}}\right)\varphi_{k_{0}}^{j_{0}}\left(x\right)+\overset{J-1}{\underset{j=j_{0}\;}{\sum }}\underset{k\in Z^{j}}{\sum }\alpha_{k}^{j}\psi_{k}^{j}\left(x\right), \end{array}$$

where *Z* ^*j*^≔0,1, 2,…, 2^*j*^, and the interpolation wavelet transform coefficient can be denoted as:

(11) αkj=u(xj+12k+1) −  [∑k0=02j0u(xj0k0)φj0k0(xj+12k+1)+∑j1=j0 j−1∑k1=02j1−1αj1k1ψj1k1(xj+12k+1)]=∑n=02J[Rj+1,J2k+1,n−∑k0=02j0Rj0,Jk0,nφj0k0(xj+12k+1)]u(xJn) −  ∑n=0 2J∑j1=j0 j−1∑k1=02j1−1αj1k1ψj1k1(xj+12k+1),

where, 0 ≤ *j* ≤ *J* − 1, *k* ∈ *Z* ^*j*^,  *n* ∈ *Z* ^*J*^, and *R* is the restriction operator defined as:

(12) $$\begin{array}{c} R_{i,m}^{l,j}=\{\begin{array}{cc} 1, & x_{i}^{l}=x_{m}^{j},\\ 0, & \text{others}. \end{array} \end{array}$$

Suppose

(13) $$\begin{array}{c} \alpha_{k}^{j}=\overset{2^{J}}{\underset{n=0}{\sum }}C_{k,n}^{j,J}u\left(x_{n}^{J}\right). \end{array}$$

Substituting (13) into (11), we obtain

(14) Ck,nj,J=R2k+1,nj+1,J−∑k0=02j0Rk0,nj0,Jφk0j0(x2k+1j+1)−∑j1=j0 j−1∑k1=02j1−1Ck1,nj1,Jψk1j1(x2k+1j+1).

If *j* = *j*_0_, then

(15) $$\begin{array}{c} C_{k,n}^{j,J}=R_{2k+1,n}^{j+1,J}-\overset{2^{j_{0}}}{\underset{k_{0}=0}{\sum }}R_{k_{0},n}^{j_{0},J}\varphi_{k_{0}}^{j_{0}}\left(x_{2k+1}^{j+1}\right). \end{array}$$

Substituting the restriction operator (12) and the wavelet transform coefficient (13) into (10), the approximate expression of the solution *u*(*x*) can be obtained as:

(16) uJ(x)=∑i∈ZJ(∑k0=02j0Rk0,nj0,Jφk0j0(x2k+1j+1)  −∑j1=j0 j−1∑k1=02j1−1Ck1,nj1,Jψk1j1(x2k+1j+1))u(xiJ).

According to the definition of the interpolation operator (9), it's easy to obtain the expression of the interpolation operator as follows:

(17) $$\begin{array}{c} I_{i}\left(x\right)=\overset{2^{j_{0}}}{\underset{k_{0}=0}{\sum }}R_{k_{0},i}^{j_{0},J}\varphi_{k_{0}}^{j_{0}}\left(x\right)+\overset{J-1}{\underset{j=j_{0}}{\sum }}\underset{k\in Z^{j}}{\sum }C_{k,i}^{j,J}\psi_{k}^{j}\left(x\right). \end{array}$$

The corresponding *m*-order derivative of the interpolation operator is

(18) $$\begin{array}{c} D_{i}^{\left(m\right)}\left(x\right)=\overset{2^{j_{0}}}{\underset{k_{0}=0}{\sum }}R_{k_{0},i}^{j_{0},J}\varphi_{k_{0}}^{j_{0}(m)}\left(x\right)+\overset{J-1}{\underset{j=j_{0}}{\sum }}\underset{k\in Z^{j}}{\sum }C_{k,i}^{j,J}\psi_{k}^{j(m)}\left(x\right). \end{array}$$

Substitute (17) and (18) into the nonlinear PDEs, and it can be changed to a system of nonlinear ODEs, the approximate analytical solution of which can be obtained with the variational iteration method (VIM).

## 3. Coupling Technique of VIM and Sparse Grid Method for Nonlinear PDEs

As mentioned above, the multilevel interpolation operator is independent of the basic functions; that is, any basis function with the interpolation property can be employed in (17) directly. But the basis function without the *m*th order derivative cannot be employed in (18) directly. In this section, we just consider the parabolic PDEs with the second order derivative as follows:

(19) −∂∂x(p(x)∂u∂x)+r(x)∂u∂x+q(x)u=∂u∂t+f(x),x∈[a,b], (x,t)∈D,u(a,0)=α,  p(b)∂u(b,0)∂x+g(b)u(b,0)=β,

where *D* is the definition domain in *x*-*t* plane.

Therefore, there are two cases that will be discussed in detail in the following. One is that the basic function to be employed in (7) has second-order derivative; the other aims at the Faber-Schauder scaling function.

### 3.1. Basis Function with *C* ^2^ Continuity

Substituting (16) into (19), it is easy to obtain the nonlinear matrix differential equations as follows:

(20) $$\begin{array}{c} L\left(\dot{V},V,t\right)+N\left(\dot{V},V,t\right)=G\left(t\right), \end{array}$$

where *L* is a linear operator, *N* a nonlinear operator, and **G**(*t*) is an inhomogeneous term, **V** is an *n*-dimensional unknown vector, and dot stands for the differential with respect to time variable *t*. For convenience, (20) can be rewritten as:

(21) $$\begin{array}{c} \dot{V}-HV-F\left(\dot{V},V,t\right)=0 \end{array}$$

**H** is a given *n* × *n* constant matrix, and $F(\dot{V},V,t)$ is an *n*-dimensional nonlinear external force vector.

According to VIM, we can write down a correction functional as follows:

(22) Vn+1(t)=Vn(t)+∫0tλ⌊V˙n(τ)−HVn(τ)        −F(V~˙n,V~n,τ)⌋dτ,

where ***λ*** is a general Lagrange vector multiplier [23] which can be identified optimally via the variational theory. The subscript *n* denotes the *n*th approximation and ${\tilde{V}}_{n}$ is considered as a restricted variation [24–27]; that is, $\delta {\tilde{V}}_{n}=0$.

Using VIM, the stationary conditions of (22) can be obtained as follows:

(23) $$\begin{array}{c} \lambda^{\prime }+\lambda H=0,\\ {1+\lambda \left(\tau \right)|}_{\tau =t}=0. \end{array}$$

The Lagrange vector multiplier can therefore be readily identified,

(24) $$\begin{array}{c} \lambda \left(\tau \right)=-e^{H(t-\tau )}. \end{array}$$

As a result, we obtain the following iteration formula:

(25) Vn+1(t)=Vn(t)−∫0teH(t−τ)⌊V˙n(τ)−HVn(τ)−F(V~˙n,V~n,τ)⌋dτ.

According to VIM, we can start with an arbitrary initial approximation that satisfies the initial condition. So we take the exact analytic solution of $\dot{V}-HV=0$ as the initial approximation; that is,

(26) $$\begin{array}{c} V_{0}\left(t\right)=e^{Ht}A, \end{array}$$

where **A** is the given initial value vector.

Substituting (26) into (25) and after simplification, we have

(27) $$\begin{array}{c} V_{n+1}\left(t\right)=V_{n}\left(t\right)+\int_{0}^{t}e^{H(t-\tau )}F\left({\dot{\tilde{V}}}_{n},{\tilde{V}}_{n},\tau \right)d\tau . \end{array}$$

According to the theory of matrices, the analytical expression of the external force $F\left({\dot{\tilde{V}}}_{n},{\tilde{V}}_{n},\tau \right)$ is required now, but it is not always available except $F\left({\dot{\tilde{V}}}_{n},{\tilde{V}}_{n},\tau \right)$ is a constant vector **f**; that is,

(28) $$\begin{array}{c} F\left({\dot{\tilde{V}}}_{n},{\tilde{V}}_{n},\tau \right)=f. \end{array}$$

The integration term of (15) is

(29) $$\begin{array}{c} \int_{0}^{t}e^{H(t-\tau )}fd\tau =\left(e^{Ht}-I\right)H^{-1}f, \end{array}$$

where the exponential matrix *e* ^**H***t*^ can be calculated accurately in PIM, and **I** is a unit matrix. Substituting (29) into (27), we obtain the variational iteration formula of the matrix differential equation as follows:

(30) $$\begin{array}{c} V_{n+1}\left(t\right)=V_{n}\left(t\right)+\left(e^{Ht}-I\right)H^{-1}f. \end{array}$$

*e* ^**H***t*^ can be solved exactly by means of the precise integration method (PIM) [28].

### 3.2. Basis Function with *C* ^1^ Continuity

Faber-Schauder scaling function is the typical basis with *C* ^1^ continuity. For convenience to construct the variational equation, the parameter *t* should be discretized as *t*_0_, *t*_1_, *t*_2_,…, *t*_*m*_, *t*_*m*_ + 1,…, where *t*_0_ = 0, *t*_*m*_ = *m*Δ*t*. Then, ∂*u*/∂*t* can be approximated as:

(31) $$\begin{array}{c} \frac{\partial u}{\partial t}\approx \frac{1}{\Delta t}\left[u\left(x,m\Delta t\right)-u\left(x,\left(m-1\right)\Delta t\right)\right]. \end{array}$$

Substituting above equation into (19), we obtain

(32) −ddx(p(x)dudx)+r(x)dudx+q(x)u=F(x),x∈[a,b], (x,t)∈D,u(a,0)=α,  p(b)du(b,0)dx+g(b)u(b,0)=β,F(x)=f(x)+1Δt[u(x,mΔt)−u(x,(m−1)Δt)].

Obviously, it is the initial-boundary elliptic PDEs. Using the virtual displacement theory, the variation equation can be obtained as:

(33) $$\begin{array}{c} a\left(u,v\right)=G\left(v\right),\;u\in H_{E}^{1}\left(a,b\right),\;\;\forall v\in H_{0E}^{1}\left(a,b\right), \end{array}$$

where

(34) HE1(a,b)≔{u∈H1(a,b) ∣ u(a)=α},H0E1(a,b)≔{v∈H1(a,b) ∣ v(a)=0},a(u,v)≔∫ab[p(x)dudxdvdx+r(x)dudxv+q(x)uv]dx+g(b)u(b)v(b),G(v)≔∫abF(x)v(x)dx+βv(b),

*H* ^1^(*a*, *b*) is the Sobolev space.

According to the interpolation wavelet transform theory, the variables *u* and *v* can be approximated as:

(35) $$\begin{array}{c} u\left(x,t\right)=\sum_{k=0}^{2^{j_{0}}}u\left(x_{j_{0},k}\right)w_{k}^{j_{0}}\left(x\right)+\sum_{j=j_{0}\;}^{J-1}\sum_{k=0}^{2^{j}-1}\alpha_{j,k}\left(t\right)w_{2k+1}^{j+1}\left(x\right)\\ v\left(x,t\right)=\sum_{k=0}^{2^{j_{0}}}v\left(x_{j_{0},k}\right)w_{k}^{j_{0}}\left(x\right)+\sum_{jv=j_{0}\;}^{J-1}\sum_{kv=0}^{2^{jv}-1}\alpha_{jv,kv}\left(t\right)w_{2kv+1}^{jv+1}\left(x\right). \end{array}$$

The first-order derivatives are

(36) ddxu(x,t)=∑k=02j0u(xj0,k)(wkj0(x))′+∑j=j0J−1∑k=02j−1αj,k(t)(w2k+1j+1(x))′,ddxv(x,t)=∑k=02j0v(xj0,k)(wkj0(x))′+∑jv=j0J−1∑kv=02jv−1αjv,kv(t)(w2kv+1jv+1(x))′,

respectively. Substituting (35)-(36) into (32), the sparse method for the parabolic PDEs based on the Faber-Schauder scaling function will be obtained. The system of ODEs can be solved exactly by means of the precise integration method (PIM).

## 4. Dynamic Choice Scheme on the External Grid Points

Combining the multilevel interpolation operator with the threshold scheme, it is easy to obtain the sparse inner grid points dynamically. Any adaptive method can capture the steep gradient appearing in the solution; that is, the inner grid points can concentrate around the larger gradient points adaptively. The PDEs in engineering are always defined in the finite domain, so the boundary condition can usually change the smoothness of solution around the boundary. This results in that more grid points around the boundary contribute to the solution and increase the calculation amount. The reasonable choice of the external grid points can decrease the boundary effect and improve the precision of the solution. In this section, we try to give a dynamic choice scheme of the external grid points, which is deduced from concept of the interval interpolation wavelet and is different from it.

### 4.1. Construction of the Interval Interpolation Wavelet

In general, the interpolation basis functions defined in interval can be represented as:

(37) ωjk ={ω(2jx−k)+∑n=−L+1−1ankω(2jx−n),k=0,…,Lω(2jx−k),k=L+1,…,2j−L−1ω(2jx−k)+∑n=2j+12j+L−1bnkω(2jx−n),k=2j−L,…,2j,

where

(38) $$\begin{array}{c} a_{nk}=\prod_{\begin{array}{c} i=L-1\\ i\neq k \end{array}}^{-1}\frac{x_{j,n}-x_{j,i}}{x_{j,k}-x_{j,i}},\;\;b_{nk}=\prod_{\begin{array}{c} i=2^{j}+1\\ i\neq k \end{array}}^{2^{j}+1+L}\frac{x_{j,n}-x_{j,i}}{x_{j,k}-x_{j,i}},\\ x_{j,k}=k\frac{x_{\max}-x_{\min}}{2^{j}},\;k\in \mathbb{Z}, \end{array}$$

where *L* is the amount of the external collocation points; the amount of discrete points in the definition domain is 2^*j*^ + 1  (*j* ∈ *Z*); [*x*_min⁡_, *x*_max⁡_] is the definition domain of the approximated function.

Equations (37) and (38) show that the interval wavelet is derived from the domain extension. The supplementary discrete points in the extended domain are called external points. The value of the approximated function at the external points can be obtained by Lagrange extrapolation method. Using the interval wavelet to approximate a function, the boundary effect can be left in the supplementary domain; that is, the boundary effect is eliminated in the definition domain.

According to (37) and (38), the interval wavelet approximant of the function *f*(*x*)*x* ∈ [*x*_min⁡_, *x*_max⁡_] can be expressed as

(39) $$\begin{array}{c} f_{j}\left(x\right)=\sum f_{j}\left(x_{n}\right)W_{j}\left(2^{j}x-n\right),\\ x_{n}=x_{\min}+n\frac{x_{\max}-x_{\min}}{2^{j}}, \end{array}$$

*f*_*j*_(*x*_*n*_) is the given value at the discrete point *x*_*n*_. At the external points, *f*_*j*_(*x*_*n*_) can be calculated by extrapolation method; that is,

(40) fj(xn)={∑k=0L−1(fj(xk)∏i=0i≠kL−1xn−xixk−xi),n=−1,…,−L∑k=2j−L+12j(fj(xk)∏i=2j−L+1k≠i2jxn−xixk−xi),n=2j+1,…,2j+L.

So the interval wavelet approximant of *f*(*x*) can be rewritten as:

(41) fj(x)=∑n=−L−1(∑k=0L−1fj(xk)∏i=0L−1xn−xixk−xi)ω(2jx−n)+∑n=02jfj(xk)ω(2jx−n)+∑n=2j+12j+L(∑k=2j−L2jfj(xk)∏i=2j−L2jxn−xixk−xi)ω(2jx−n).

Let

(42) $$\begin{array}{c} {\text{LS}}_{L}\left(x_{n}\right)=\sum_{k=0}^{L-1}f_{j}\left(x_{k}\right)\prod_{i=0}^{L-1}\frac{x_{n}-x_{i}}{x_{k}-x_{i}},\\ {\text{LE}}_{L}\left(x_{n}\right)=\sum_{k=2^{j}-L}^{2^{j}}f_{j}\left(x_{k}\right)\prod_{i=2^{j}-L}^{2^{j}}\frac{x_{n}-x_{i}}{x_{k}-x_{i}} \end{array}$$

then

(43) fj(x)=∑n=−L−1LSL(xn)ω(2jx−n)+∑n=02jfj(xk)ω(2jx−n)+∑n=2j+12j+LLEL(xn)ω(2jx−n).

LS_*L*_(*x*_*n*_) and LE_*L*_(*x*_*n*_) correspond to the left and the right external points, respectively. They are obtained by Lagrange extrapolation using the internal collocation points near the boundary. So, the interval wavelet's influence on the boundary effect can be attributed to Lagrange extrapolation. It should be pointed out that we did not care about the reliability of the extrapolation. The only function of the extrapolation is enlarging the definition domain of the given function which can avoid the boundary effect occurring in the domain. Therefore, we can discuss the choice of *L* by means of Lagrange inner- and extrapolation error polynomial as follows:

(44) RL(x)=f(L+1)(ξ)(L+1)!∏i=0L(x−xi),for  some  ξ  between  x,x0,…,xL.

Equation (44) indicates that the approximation error is both related to the smoothness and the gradient of the original function near the boundary. Setting different *L* can satisfy the different error tolerance requirement.

### 4.2. Dynamic Choice Scheme of External Points in Sparse Grids Approach

This scheme is made up with 2 steps. First, the Newton interpolation operator is employed instead of the traditional Lagrange interpolation. Second, both of the error tolerance and condition number are taken as the termination procedure of dynamic choice of external grid points. We will discuss it in detail in this section.

In order to construct the dynamic choice scheme of external grid points, the Newton interpolation theory should be introduced instead of the traditional Lagrange interpolation theory. It is well known that the Newton interpolation is equivalent with Lagrange interpolation, but the Lagrange interpolation algorithm has no inheritance which is the key feature of Newton interpolation. So, the advantage of Newton interpolation method is that the basis function does not need to be recalculated as one point is added except only one more term is needed to be added, which reduces the number of compute operation, especially the multiplication.

The expression of Newton interpolation can be written as:

(45) Nn(x)=f(x0)+(x−x0)f(x0,x1)+(x−x0)(x−x1)f(x0,x1,x2)+⋯+(x−x0)(x−x1),…,(x−xn−1)×f(x0,x1,…,xn),

substituting the Newton interpolation instead of the Lagrange interpolation into (43), which can be rewritten as:

(46) fj(x)=∑n=−L−1(NSL(xn))ω(2jx−n)+∑n=02jfj(xn)ω(2jx−n)+∑n=2j+12j+L(NEL(xn))ω(2jx−n),

where

(47) NSL(xn)=f(x0)+(xn−x0)f(x0,x1)+(xn−x0)(xn−x1)f(x0,x1,x2)+⋯+(xn−x0)(xn−x1),…,(xn−xL−1)×f(x0,x1,…,xL),NSR(xn)=f(x2j)+(xn−x2j)f(x2j,x2j−1)+(xn−x2j)(xn−x2j−1)f(x2j,x2j−1,x2j−2)+⋯+(xn−x2j)(xn−x2j−1),…,(xn−x2j−L)×f(x2j,x2j−1,…,x2j−L).

It is well known that the Newton interpolation is equivalent to the Lagrange interpolation. The corresponding error estimation can be expressed as:

(48) $$\begin{array}{c} R_{n}\left(x\right)=\left(x-x_{0}\right)\left(x-x_{1}\right),\ldots ,\left(x-x_{n}\right)f\left(x,x_{0},\ldots ,x_{n}\right). \end{array}$$

And the simplest criterion to terminate the dynamic choice on *L* is |*R*_*n*_(*x*)| ≤ Ta (Ta is the absolute error tolerance). Obviously, it is difficult to define Ta which should meet with the precision requirement of all approximated curves. In fact, the difference coefficient *f*(*x*, *x*_0_,…, *x*_*n*_) can be used directly as the criterion; that is,

(49) $$\begin{array}{c} \left|f\left({x,x}_{0},\ldots ,x_{n}\right)\right|<\varepsilon . \end{array}$$

As mentioned above, once the curves with lower-order smoothness are approximated by higher-order polynomial expression, the errors will become bigger on the contrary. In fact, even if the *L* is infinite, the computational precision cannot be satisfied except increasing computational complexity. To avoid this, we design the termination procedure of dynamic choice about *L* as follows:

 If  *f*(*x*_0_, *x*_1_) < Ta, then  *L* = 1
 elseif  *f*(*x*_0_, *x*_1_, *x*_2_) < Ta
 or  *f*(*x*_0_, *x*_1_, *x*_2_) < *f*(*x*_0_, *x*_1_), then  *L* = 2
 elseif  *f*(*x*_0_, *x*_1_, *x*_2_, *x*_3_) < Ta
 or  *f*(*x*_0_, *x*_1_, *x*_2_, *x*_3_) < *f*(*x*_0_, *x*_1_, *x*_2_), then  *L* = 3
  ⋮ 

In the field of numerical analysis, the condition number of a function with respect to an argument measures how much the output value of the function can change for a small change in the input argument. This is used to measure how sensitive a function is to changes or errors in the input, and how much error in the output results from an error in the input. There is no doubt that the choice of *L* can change the condition number of the system of algebraic equations discretized by the wavelet interpolation operator or the finite difference method. Therefore, the choice of *L* should take the condition number into account. In fact, if the condition number cond(*A*) = 10^*k*^, then you may lose up to *k* digits of accuracy on top of what would be lost to the numerical method due to loss of precision from arithmetic methods. According to the general rule of thumb, the choice should follow the rule as follows:

(50) $$\begin{array}{c} \frac{\text{Cond}\left(A_{L+1}\right)}{\text{Cond}\left(A_{L}\right)}<10. \end{array}$$

The computational complexity of interpolation calculation is not proportional to the increasing points. The former is mainly up to the computation amount of (*x* − *x*_0_)(*x* − *x*_1_),…, (*x* − *x*_*n*_) and the derivative operations. Obviously, according to (9), the increase in computational complexity is *O*(*L* ^3^) when the number of extension points *L* increases by 1. But the computational complexity of adaptively increasing collocation points is related to the different basis functions. For the basis with compact support property such as Daubechies wavelet and Shannon wavelet, the value of *L* is impossible to be infinite. For Haar scaling function which has no smoothness property, *L* can be taken as 0 at most since it does not need to be extended. For Faber-Schauder wavelet, *L* can be taken as 1 at most. For Daubechies wavelet, *L* can be taken as different values according to the order of vanishing moments, but it must be finite. For the wavelets without compact support property, *L* can take value dynamically, such as Shannon wavelet. The computational complexity of increasing points is mainly dependent on the basis function of itself.

## 5. Numerical Experiments

### 5.1. Dynamic Choice of the Sparse Grid Points

In order to test the adaptability of the sparse grid approach proposed in this paper, the Faber-Schauder and Shannon scaling function, the autocorrelation function of Daubechies scaling functions, are taken as the basis, respectively. Faber-Schauder scaling function has first-order derivative and Daubechies scaling function has second derivative, so both of the dynamic choice schemes will be tested.

**Example 1**

Burger equation with Dirichlet boundary conditions.

**Figure 1:**
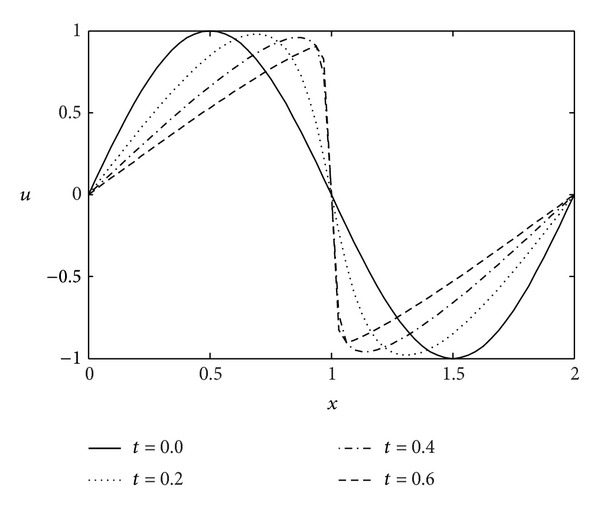
Analytical solutions of the Burgers equation at different times (*t* = 0,0.4,0.6).

As a test problem for the numerical algorithm described in the previous section, we will consider Burgers equation as follows:

(51) ∂u∂t+u∂u∂x=1Re∂2u∂x2, x∈[0,2]

with initial and boundary conditions

(52) $$\begin{array}{c} u\left(x,0\right)=\sin\left(\pi x\right)\\ u\left(0,t\right)=u\left(2,t\right)=0, \end{array}$$

where *t* represents the time and *Re* denotes the Reynolds number. With the increasing of the value of *Re*, the solution develops into a saw-tooth wave at the origin point. The gradient at the origin reaches its maximum value. Therefore, the performance of a numerical method is often judged by its ability to resolve the large gradient region that develops in the solution, which is shown in Figure 1.

Using the difference coefficient to approximate the partial differential operator ∂*u*/∂*t*, the Burgers equation becomes

(53) −1Red2u(x,m.Δt)dx2+u(x,(m−1)Δt)·du(x,mΔt)dx +1Δt[u(x,mΔt)−u(x,(m−1)Δt)]=0,u(0,mΔt)=u(2,mΔt)=0, m=1,2,….

According to the virtual displacement theory, the variational form of the Burgers equation can be represented as:

(54) ∫02εdu(x,m·Δt)dx·dv(x)dxdx +∫02[u(x,(m−1)Δt)du(x,m·Δt)dt    +1Δtu(x,m·Δt)]v(x)d(x) =∫021Δtu(x,(m−1)Δt)vdx.

This can be solved by means of (35)-(36).

In the experiments, the Reynolds number *Re* = 1000, and the time step *τ* = 0.001.

**Figure 2:**
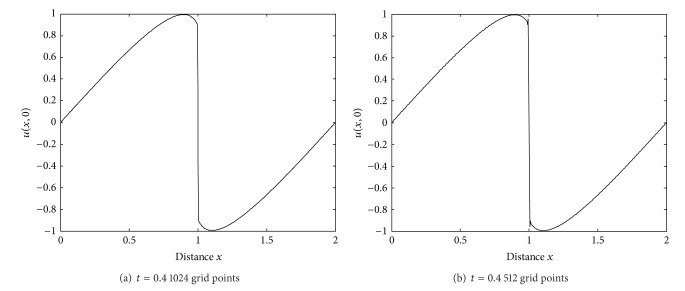
Numerical solution obtained by the finite difference method.

The numerical results showed in Figure 2 are obtained by the finite difference method. As the amount of the even discrete points is taken as 512, the Gibbs phenomena appeared at *x* = 1 where exists a steep slope in the solution (Figure 2(b)). Increasing the discrete points can restrict the Gibbs phenomena (Figure 2(a)).

**Figure 3:**
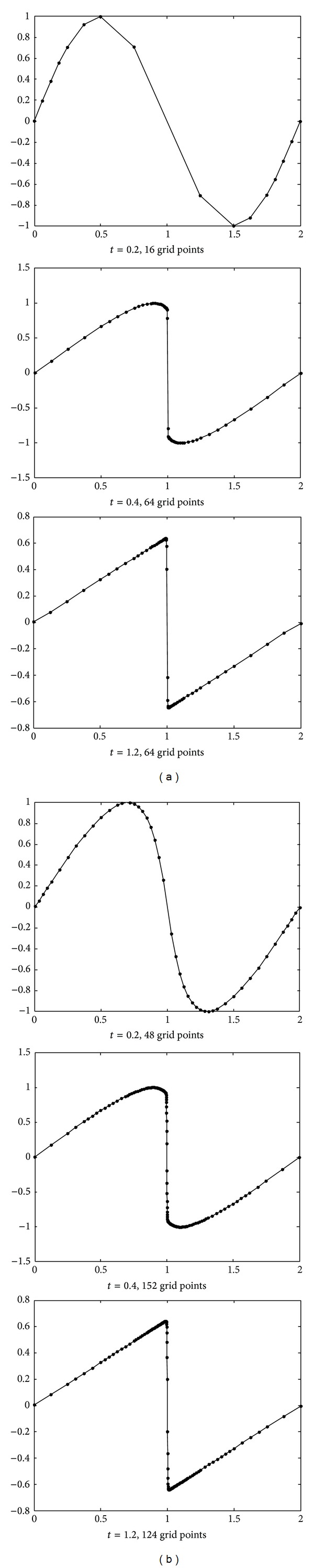
Solution evolution of Burgers equation with *Re* = 1000.

Figure 3 illustrates the performance of the sparse solution method on this example by comparison of the sparse solution and the true solution produced using a standard fully resolved method (finite difference method).

With the increasing of the parameter *t*, the gradient of the solution at the point *x* = 1 becomes more and more large. Any of the Faber-Schauder scaling function and the auto-correlation function of the Daubechies scaling function is taken as the basis functions, the sparse method can capture the steep slope appeared in the solution effectively. This shows that more and more grid points concentrate around the point *x* = 1. However, the maximum of the gradient at *x* = 1 appeared as *t* = 0.4, the 1024 coefficients used in the true solution, only 64 with Faber-Schauder basis and 152 with Daubecies basis, are retained in the sparse solution (about 6.25% and 14.84%), respectively. Begin with *t* = 0.4, the gradient value of the solution at *x* = 1 becomes smaller and smaller with the increasing of *t*. The amount of the sparse grid points also decreases with the increasing of *t* accordingly. This is illustrated in Figure 3. The adaptability of the proposed sparse method is helpful to improve the efficiency and the calculation precision of the algorithm.

**Figure 4:**
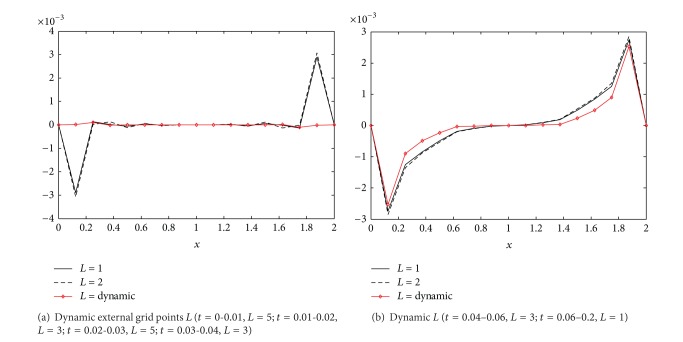
The influence of the condition number to the error (*Re* = 1000, *j* = 7).

Besides, we also noticed that the condition number of Burger equation from Table 1 varies with the change of *j*, *Re*, and the time step *τ*. In fact, the condition number relates closely with the sparse grid points. As *Re* and *j* are smaller, the steep gradient will not appear in the solution and the grid points are sparse. In this case, the condition number is smaller and will not destroy the numerical precision apparently. On the contrary, if the *Re*, *j*, and the time step are larger, the steep slope appearing in the solution and the error brought from the larger time step will bring more grid points adaptively into the algorithm. This will deduce that the condition number becomes larger. It has been mentioned in Section 4.2 that the larger condition number can decrease the calculation precision greatly. Table 1 shows that the condition number (*L* = 2) increases more rapidly than *L* = 1 with the increase of *j* and *Re*. This also can be illustrated in Figure 4.

Figure 4 illustrates that the external grid points change with the development of *t* dynamically. As *t* ≤ 0.04 (Figure 4(a)), the solution function is smooth and the condition number is smaller. The approach can take more external grid points dynamically to improve the precision. As *t* > 0.04 (Figure 4(b)), the steep slope is appearing in the solution and the condition number is increasing. In this case, the increase of the external grid points cannot improve the precision anymore. In fact, this explained the reason why we construct the dynamic choice scheme of external grid points to some extent.

### 5.2. Comparison between the Dynamic Choice Scheme and the Wavelet Collocation Method

**Example 2**

Consider the Heat equation

(55) ∂u∂t=∂2u∂2x+ex+2t, x∈[0,1],  0≤t≤T,      

with the initial and boundary conditions

(56) $$\begin{array}{c} u\left(x,0\right)=e^{x},\;\;\frac{\partial u\left(0,t\right)}{\partial x}=e^{2t},\;\;\frac{\partial u\left(1,t\right)}{\partial x}=e^{1+2t}, \end{array}$$

where *t* denotes the time parameter.

Let *I*_*i*_(*x*) and *D*_*i*_(*x*) denote the interpolation operator and the corresponding derivative; according to the classical collocation approach, the approximating formulation *u*_*J*_(*x*) of a function *u*(*x*) can be written as:

(57) $$\begin{array}{c} u_{J}\left(x\right)=\sum_{i\in Z^{c}}I_{i}\left(x\right)u_{Ji}. \end{array}$$

Substituting (57) into (55) leads to a system of nonlinear ordinary differential equations as follows:

(58) $$\begin{array}{c} \sum_{n\in Z^{c}}^{}u_{J}\left(x_{n},t\right)D_{n}^{\prime \prime }\left(x_{k}\right)+\exp\left(x_{k}+2t\right)=\frac{\partial u_{j}\left(x_{k},t\right)}{\partial t}, \end{array}$$

where *k* ∈ *Z*_*c*_. The corresponding vector expression is

(59) $$\begin{array}{c} \frac{\partial }{\partial t}V_{J}=M_{0}V_{J}+F\left(t\right). \end{array}$$

The corresponding Neumann boundary condition can be expressed as:

(60) $$\begin{array}{c} M_{1}\left(1,1\right)V_{J}\left(1\right)+\sum_{i=2}^{2^{J}}M_{1}\left(1,i\right)V_{J}\left(i\right)=e^{2t},\\ M_{1}\left(2^{J},2^{J}\right)V\left(2^{J}\right)+\sum_{i=2}^{2^{J}}M_{1}\left(2^{J},i\right)V_{J}\left(i\right)=e^{1+2t}, \end{array}$$

where

(61) $$\begin{array}{c} V_{J}={\left(u_{J}\left(x_{0},t\right),u_{J}\left(x_{1},t\right),\ldots ,u_{J}\left(x_{2^{J}},t\right)\right)}^{T},\\ F\left(t\right)={\left(\exp\left(x_{0}+2t\right),\exp\left(x_{1}+t\right),\ldots ,\exp\left(x_{2^{J}}+2t\right)\right)}^{T},\\ M_{0}\left(k,n\right)=m_{k,n}^{0}=D_{n}^{\prime \prime }\left(x_{k}\right),\;k,n\in Z^{c},\\ M_{1}\left(k,n\right)=m_{k,n}^{1}=D_{n}^{\prime }\left(x_{k}\right),\;k,n\in Z^{c}. \end{array}$$

**Figure 5:**
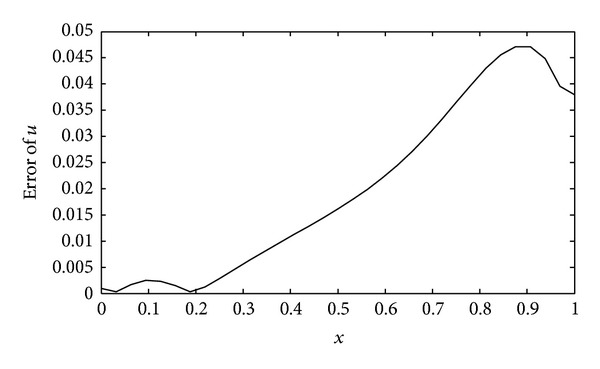
Error of the solution with the dynamic grid approach (*T* = 0.2, *τ* = 0.01, *j* = 5).

**Figure 6:**
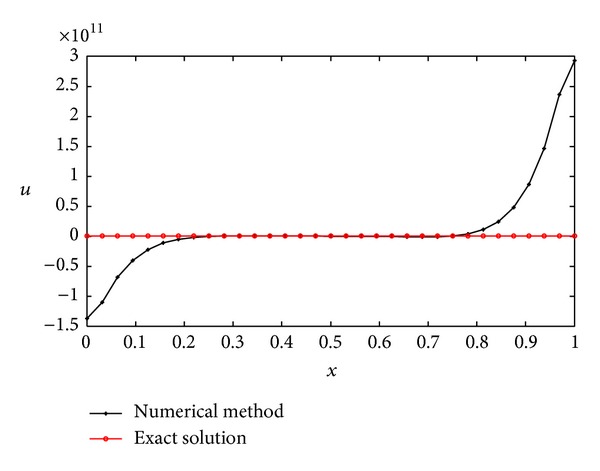
Numerical solution with interval wavelet method (*L* = 4, *T* = 0.2, *τ* = 0.01, *j* = 5).

**Figure 7:**
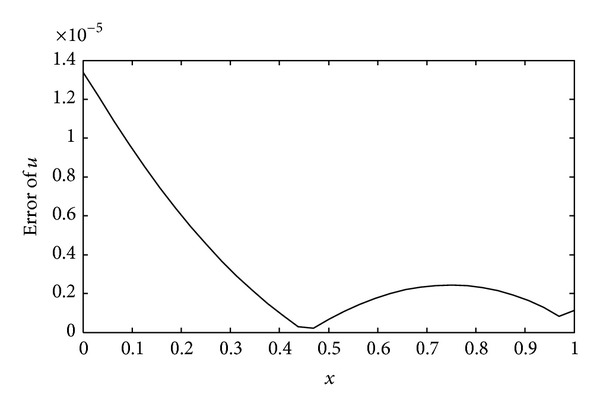
Error of the solution with the dynamic sparse grid approach (*j* = 5, *T* = 0.2, and *τ* = 0.00001).

**Figure 8:**
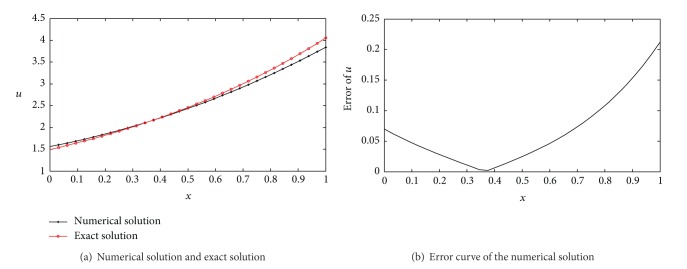
Numerical solution and error curves with interval wavelet Runge-Kutta method (*L* = 4, *T* = 0.2, *τ* = 0.00001, *j* = 5).

Equations (59)-(60) can be solved by the VIM and PIM. In the following, we will take heat equation as examples to illustrate the effectiveness of the algorithm proposed in this paper. The Shannon scaling function is employed to be the basis function. The exact analytical solution of (55) is *u*(*x*, *t*) = *e* ^*x*+2*t*^. Obviously, the solution function's infinite order derivative exists for all *x* in the definition domain.

(*1) Comparison between the Dynamic Choice Scheme and the Static Interval Wavelet. *Let *T* = 0.2 and let *τ* = 0.01. The computational error curve of the dynamic sparse grid approach is shown in Figure 5. The maximum of the absolute error is 0.0471, which occurs near the right boundary. This shows that the bigger gradient of the solution can cause bigger error. The dynamic *L* and the iteration times at the same *L* value are shown in Table 2. The value of *L* varies between 2, 3, and 4, and the iteration times at *L* = 4 is much more than *L* equaling 2 or 3. So, we take *L* = 4 in the static interval wavelet PIM to solve the heat equation in the same parameters with the dynamic scheme. The numerical solution is shown in Figure 6. Obviously, the error is too big that the algorithm is invalid. There are many reasons that can lead to this result such as the smoothness of the solution and the nonlinear term in the PDEs. As the time step *τ* = 0.00001, the error curve was shown in Figure 7. The dynamic *L* and the iteration times at the same *L* value are shown in Table 3. With the decreasing of the time step, the influence of the nonlinear term on PDEs becomes smaller and smaller. The biggest errors of both dynamic and static interval wavelet PIM are 1.3388 × 10^−5^. This shows that the construction of dynamic grid approach is necessary for nonlinear PDEs with Neumann boundary conditions. 

(*2) Comparison between the VIM and PIM and Runge-Kutta Method for Time-Domain Integration. *The numerical solution and error curves with the VIM and PIM and Runge-Kutta method are shown in Figure 8. It is obvious that the calculation precision of VIM and PIM (Figure 5) is better than Runge-Kutta method. It should be pointed out that Runge-Kutta is not sensitive to the time step *τ* (Table 4) compared with VIM and PIM. One of the most important reasons is that the nonlinear term in PDEs was integrated with explicit format in VIM and PIM, and implicit format was employed in Runge-Kutta method.

## 6. Conclusions

The multilevel interpolation operator constructed in this paper is independent of the basis. Although Faber-Schauder scaling function has no second-order derivative, it still can be the basis employed in the multiscale interpolation operator to solve the Burgers equation, while only retaining important nodes. The reduced dynamics created by the sparse projection property can dynamically capture the true phenomena exhibited by the solution. This sparse projection amounts to a shrinkage of the coefficients of the updated solution at every time step. Compared with the finite difference method, the retained coefficients are less than 10% in the sparse solution of the Burgers equation.

The dynamic sparse grid approach, which is constructed by combining the multiscale interpolation operator and the variational iteration method, is able to choose both of the internal and external grid points based on the gradient and the smoothness of the solution, the condition number of the PDEs, and the error tolerance dynamically. This property is good suit to the PDEs with Neumann boundary conditions. It can eliminate the boundary effect efficiently. With regard to the accuracy and time complexity of the solution in comparison with those obtained with other algorithms, the dynamic sparse grid approach constructed in this paper is more reasonable. The numerical experiments illustrate that it is necessary to construct the dynamic sparse grid approach for the nonlinear PDEs with Neumann boundary conditions and Dirichlet boundary conditions.

## Figures and Tables

**Table 1:** Condition number of the Fokker-Planck equation.

*j*	Re	*τ* = 0.0001			*τ* = 0.00001		
		*L* = 0	*L* = 1	*L* = 2	*L* = 0	*L* = 1	*L* = 2
4	10	8.9742	6.3786	12.1637	1.56319	1.9723	2.3335
	100	91.8021	49.1826	186.9260	9.5434	11.9168	19.2971
	1000	1891.2	512.7634	3856.3	403.3223	148.8976	812.9812

7	10	40.3968	32.2256	82.2844	41.2311	4.3834	7.2188
	100	813.5472	199.1953	1245.7	81.7719	49.5538	173.4967
	1000	21987.0000	2426.8	39004	3688.30	699.4512	6917.4

10	10	298.5375	145.7761	663.4654	20.6612	18.4523	40.1116
	100	7821.7000	1217.8	14346	698.5623	212.9856	1274.6
	1000	194670	13887	523830	38421	3974.4	79248

**Table 2:** Dynamic *L* and the iteration times at the same *L* value (*j* = 5, *T* = 0.2, *τ* = 0.01).

*L*	3	2	4	2	3	2	4	2	3	2	4	2	3

Iteration times	2	1	2	1	1	1	3	1	1	1	3	1	1

**Table 3:** Dynamic *L* and the iteration times at the same *L* value (*j* = 5, *T* = 0.2, *τ* = 0.00001).

*L*	6	5	4	5	4

Iteration times	15	14	1	1	19968

**Table 4:** Error comparison between VIMand PIM and Runge-Kutta Method.

*τ*	0.1	0.01	0.001	0.0001	0.00001
VIM and PIM	0.4176	0.0471	0.0045	4.1368 × 10^−4^	1.7610 × 10^−5^
Runge-Kutta	0.1366	0.2193	0.2110	0.2122	0.2124
